# Appraising the Effects of Metabolic Traits on the Risk of Glaucoma: A Mendelian Randomization Study

**DOI:** 10.3390/metabo13010109

**Published:** 2023-01-09

**Authors:** Kai Wang, Fangkun Yang, Xin Liu, Xueqi Lin, Houfa Yin, Qiaomei Tang, Li Jiang, Ke Yao

**Affiliations:** 1 Eye Center of the Second Affiliated Hospital, School of Medicine, Zhejiang University, Hangzhou 310009, China; 2 Department of Cardiology, Ningbo First Hospital, Ningbo 315010, China; 3 Department of Biochemistry and Molecular Biology, School of Basic Medical Sciences, Hangzhou Normal University, Hangzhou 311121, China

**Keywords:** glaucoma, metabolic traits, type 2 diabetes, blood pressure, blood lipid, Mendelian randomization

## Abstract

Metabolic traits are associated with the risk of developing glaucoma in observational studies. To assess whether theses associations reflect causality, we conducted a Mendelian randomization (MR) study. Our study included up to 20,906 glaucoma cases and 438,188 controls. Genetic instruments associated with the concerned 11 exposures at the genome-wide significance level were selected from corresponding genome-wide association studies. Summary-level data for glaucoma were obtained from the UK Biobank, the GERA study, and the FinnGen consortium. Univariable and multivariable MR analyses were conducted separately in two populations. Our results showed that higher genetic liability to type 2 diabetes (T2D) was causally and independently associated with an increased risk of glaucoma (odds ratio [OR], 1.11; 95% confidence interval [CI], 1.06–1.16; *p* = 4.4 × 10^−6^). The association for T2D persisted after multivariable adjustment. In addition, higher genetically predicted systolic blood pressure (SBP), fasting glucose (FG), and HbA1c, were also suggestively associated with glaucoma risk. The OR was 1.08 (95% CI, 1.01–1.16; *p* = 0.035) for SBP, 1.24 (95% CI, 1.05–1.47; *p* = 0.011) for FG, and 1.28 (95% CI, 1.01–1.61; *p* = 0.039) for HbA1c. No evidence was observed to support the causal effects of body mass index and blood lipids for glaucoma. This study suggests a causal role for diabetes, as well as possible roles for higher SBP, FG, and HbA1c in the development of glaucoma. Further validation is needed to assess the potential of these risk factors as pharmacological targets for glaucoma prevention.

## 1. Introduction

Glaucoma is the leading cause of irreversible blindness in the world, which affects 76 million people worldwide and results in at least 8.4 million being bilaterally blind. Global prevalence of this blinding disease is projected to increase to 112 million by 2040 [1]. As glaucoma could occur asymptomatically until late in the disease when vision problems occur, loss of vision from the disease cannot be recovered or reversed. Treatment for glaucoma often involves daily eye drops, but adherence to treatment is often unsatisfactory [2,3]. Therefore, the identification of potentially modifiable risk factors for glaucoma is of great interest, so that interventions may be developed to reduce the incidence or improve the prognosis of the disease.

Large observational studies have established metabolic traits, such as type 2 diabetes (T2D) [4,5], dyslipidemia [6,7,8], hypertension [9,10], and obesity [11,12], as risk factors for glaucoma. However, the evidence from these observational findings is inconsistent, and the association is still controversial. Although two meta-analyses reviewed that diabetes increased the incidence of glaucoma, no significant correlation were found between diabetes and glaucoma-related traits in some observational studies and genetic correlation analysis after adjustment for confounding factors [4,5,13]. These are controversial and also persistent for the association of obesity and blood lipids with glaucoma, reporting either positive [6,11] or null [12] associations. More importantly, the causality of these relationships cannot be determined, as residual confounding and reverse causation may have biased the results, thus limiting the ability to make causal inferences. For example, obesity, dyslipidemia and T2D are closely interrelated, but their independent association with glaucoma is uncertain. In addition, even blood pressure has been recognized as a risk factor for glaucoma in epidemiological and clinical studies, and the causal relationship is unknown. A clear appraisal of the causality of these associations is therefore of importance in updating the primary prevention strategy for glaucoma.

In Mendelian randomization (MR) analysis, genetic variants during conception are used as instrumental variables to identify the causal association between exposure and health outcome. By using MR, the residual confounding can be minimized since genetic variants are randomly distributed at conception and unrelated to other factors. Reverse causation bias can also be avoided, as the genetic variants are unmodified by the onset or progression of the disease. Herein, we conducted a comprehensive MR analysis to evaluate the associations of multiple metabolic traits, including body mass index (BMI), systolic blood pressure (SBP), diastolic blood pressure (DBP), blood lipids, fasting glucose (FG), fasting insulin (FI), and T2D, with the risk of glaucoma. Multivariable MR analyses were also performed to detect potential confounders.

## 2. Methods

### 2.1. Study Design

**Figure 1:**
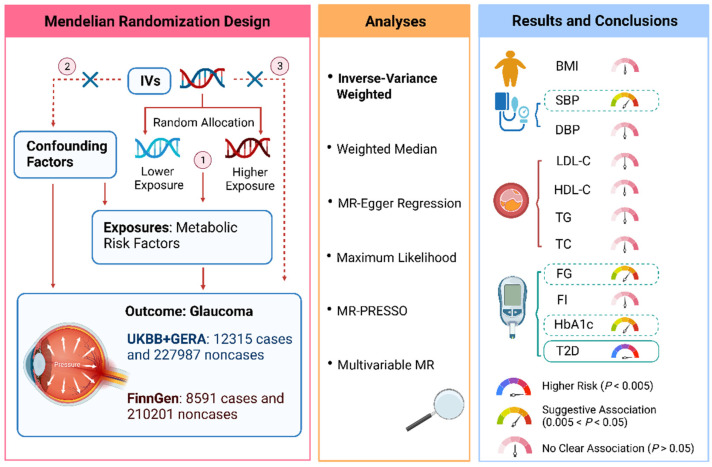
Overview of the MR study design and results. The validity of MR findings depends on the three crucial assumptions, i.e., the instrumental variables should be robustly associated with the exposure of interest (assumption 1) and not be associated with potential confounders (assumption 2) and affect the outcome only through the intermediate exposure, not through others pathways (assumption 3). To satisfy these assumptions, in addition to the main analysis (the inverse variance-weighted method), we also used multiple MR approaches as sensitivity analyses to detect and correct for pleiotropy. Abbreviations: BMI, body mass index; DBP, diastolic blood pressure; FG, fasting glucose; FI, fasting insulin; HbA1c, hemoglobin A1c; HDL-C, high-density lipoprotein cholesterol; IVs, instrumental variables; LDL-C, low-density lipoprotein cholesterol; SBP, systolic blood pressure; T2D, type 2 diabetes; TC, total cholesterol; TG, triglyceride; UKBB, UK Biobank.

We used a two-sample MR design to explore the associations of multiple metabolic risk factors with glaucoma. The overall study design is depicted graphically in Figure 1. All analyses were based on summary-level data on measures of metabolic traits and glaucoma from published genome-wide association studies (GWASs) and consortia. Appropriate ethical approval and informed patient consent can be found in the original studies.

### 2.2. Selection of the Genetic Instruments

Genetic instruments for each exposure were identified from the GWAS, including primarily of individuals of European ancestry. Detailed information on used data sources is shown in Table 1. Single nucleotide polymorphisms (SNPs) associated with BMI [14], SBP and DBP [15], low-density lipoprotein (LDL) cholesterol, high-density lipoprotein (HDL) cholesterol, triglyceride, and total cholesterol [16], FG, FI, and HbA1c [17], and T2D [18] at the genome-wide significance threshold (*p* < 5 × 10^−8^) were obtained from relevant GWASs. Then, we performed linkage disequilibrium tests for each risk factor based on 1000 Genomes reference panel (European population) using the PLINK clumping method [19]. SNPs without linkage disequilibrium (*r* ^2^ < 0.01 and clump distance > 10,000 kb) were used as instrument variables. For palindromic SNPs, if the minor allele frequency is smaller than 0.42, then this SNP was regarded as inferable [20].

### 2.3. Data Source for Glaucoma

Summary-level statistics for the association between SNPs and glaucoma were obtained from a large GWAS meta-analysis that combined the UK Biobank and the Genetic Epidemiology Research in Adult Health and Aging (GERA) [21], as well as the FinnGen consortium [22] (Table 1). The meta-analysis of UK Biobank and GERA included 12,315 glaucoma cases and 227,987 noncases, of whom 89% had European ancestry. In GERA, glaucoma cases were defined by the International Classification of Diseases, Ninth Revision (ICD-9); In the UK Biobank, the glaucoma case was assessed according to a touchscreen self-reported questionnaire. In the FinnGen consortium, we used the fifth release of the data on glaucoma, including 8591 glaucoma cases and 210,201 noncases. The glaucoma cases from the FinnGen consortium were defined according to ICD-10: H40/H42, which involved 10 specified and unspecified types of glaucoma (https://r5.risteys.finngen.fi/phenocode/H7_GLAUCOMA) (accessed on 18 August 2021).

### 2.4. Statistical Analyses

The univariable inverse-variance weighted method under a multiplicative random-effects model was used as the main statistical analyses. Estimates from the GWAS (including UK Biobank and GERA study) and FinnGen consortium were combined using the fixed-effects meta-analysis method. Additionally, we conducted multiple sensitivity analyses to evaluate the robustness of the results and check for pleiotropy, including the weighted median method, MR-Egger regression, maximum likelihood method, and Mendelian randomization pleiotropy RESidual sum and outlier (MR-PRESSO). The weighted median method is robust for invalid instruments and provides consistent causal estimates as long as over 50% of the weight in the analysis is from valid instruments [23]. MR-Egger regression can detect directional pleiotropy and generate pleiotropy-corrected estimates, but can be imprecise [24]. A non-null MR-Egger intercept suggests potential directional pleiotropy. Maximum likelihood method may provide more reliable results in the presence of measurement error in the SNP-exposure effects [25]. The MR-PRESSO method can detect potential outlier SNPs and evaluate whether removal of outliers affects the results, which also indicates potential pleiotropy [26]. The MR-PRESSO distortion test can distinguish significant differences between estimates before and after correction for outliers. Cochrane’s Q statistics were calculated to assess the heterogeneity among different genetic instruments.

In case a significant association was identified in the main analyses, we also performed the multivariable MR as a sensitivity analysis to explore whether this causal effect was robust to the adjustment of the major metabolic and lifestyle risk factors [27]. We evaluated the attenuating effects after adjusting for each factor separately.

To adjust for multiple testing, the two-sided statistical significance level was defined as 0.005 (0.05/11 exposures) according to the Bonferroni correction. A *p*-value between 0.005 and 0.05 was considered as suggestive evidence for a potential association. All statistical analyses were conducted using the TwoSampleMR (version 0.5.6) [25] and MR-PRESSO (version 1.0) [26] packages in R version 4.1.2 (R Foundation for Statistical Computing, Vienna, Austria).

## 3. Results

**Figure 2:**
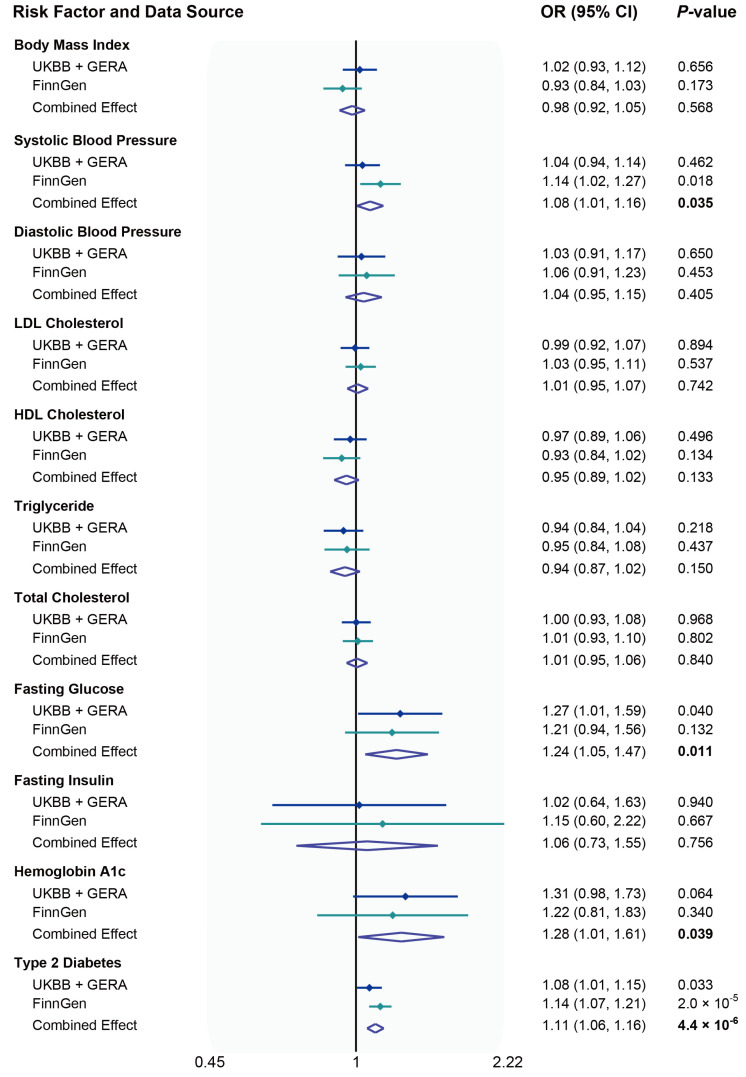
Associations of metabolic traits with glaucoma using the inverse variance-weighted method. ORs represent the associations with glaucoma, respectively: 1-SD increased in body mass index; 10-mmHg increased in blood pressure; 1-SD increased in blood lipids; 1-mmol/L increased in fasting glucose; 1-pmol/L increased in fasting insulin; 1% increased in hemoglobin A1c; 1-log unit odds of type 2 diabetes. Abbreviations: CI, confidence intervals; HDL, high-density lipoprotein; LDL, low-density lipoprotein; OR, odds ratio; UKBB, UK Biobank.

Genetically liability to T2D was associated with elevated risk of glaucoma in UK Biobank and GERA data, FinnGen consortium data, and meta-analysis (Figure 2). The combined odds ratio (OR) of glaucoma was 1.11 (95% confidence interval [CI], 1.06–1.16) for a one-unit increase in log OR of T2D (*p* = 4.4 × 10^−6^). This association was consistent in sensitivity analyses, albeit with wider CIs in the MR-Egger regression (Tables S1 and S2). After removing outliers in the MR-PRESSO analysis, the association between T2D and glaucoma persisted, and the *p* value for the distortion test were above 0.05 (Tables S1 and S2). This association was also robust in the multivariable MR analyses, but slightly attenuated after adjusting for FG (*p* = 4.6 × 10^−4^) and HbA1c (*p* = 2.0 × 10^−4^) (Table S3). There were suggestive associations of genetically predicted higher SBP (combined OR per 10 mmHg increase, 1.08; 95% CI, 1.01–1.16; *p* = 0.035), FG (combined OR per 1 mmol/L increase, 1.24; 95% CI, 1.05–1.47; *p* = 0.011), and HbA1c (combined OR per 1% increase, 1.28; 95% CI, 1.01–1.61; *p* = 0.039) with an increased risk of glaucoma (Figure 2). We did not observe any association of genetically predicted BMI, DBP, blood lipids, or FI with risk of glaucoma in the main analysis (Figure 2).

## 4. Discussion

The present MR study included up to 20,906 glaucoma cases and 438,188 controls from the UK Biobank, GERA and the FinnGen consortium, we found that T2D is independently and causally associated with the risk of glaucoma. Moreover, our results provide support for causal associations of FG, HbA1c, and SBP with risk of glaucoma. There is no evidence that blood lipids are causally associated with glaucoma.

Although T2D has been associated with glaucoma in observational studies, results were controversial. A meta-analysis including 47 cohort and case-control or cross-sectional studies showed a 5% increased risk of primary open-angle glaucoma (POAG) for each year since diabetes diagnosis, albeit significant heterogeneity across studies [4]. Another meta-analysis of 7 prospective studies also reported that diabetes increased the incidence of POAG by 36% [5]. A suggestive risk of POAG was found in a MR study from GERA cohort, including 3554 POAG cases and 39 SNPs, but inconsistency existed across sensitive analyses and a panel of genome-wide genetic biomarkers for T2D were not associated with POAG [28]. In contrast, a genetic correlation analysis demonstrated limited genetic correlation between diabetes and glaucoma-related traits after adjustment for multiple comparisons, which was consistent with individual-level data from some previous observational studies [13]. One reason for the variation in these results may be due to the biases and residual confounders from observational studies. Our MR study, which based on a large number of glaucoma disease cases, with updated T2D GWAS study including more SNPs, and with replication analysis from two populations, revealed a robust causal association between T2D and glaucoma. The results also indicated that the association was likely independent of other metabolic traits, including BMI, lipids, and blood pressure. In addition, we observed consistent positive associations for FG and HbAc1 with the risk of glaucoma, supporting the deleterious effect of hyperglycemia in the disease progression. The association between T2D and glaucoma was slightly attenuated in the multivariable MR analysis after adjustment for genetically predicted FG and HbAc1 liability, which may suggest that blood glucose status partly mediates this association. Several possible mechanisms may explain the association between T2D and glaucoma. For example, diabetes has been suggested to cause microvascular damage and vascular dysregulation of the retina and the optic disc, increasing the susceptibility of the optic nerve head to damage [29,30]. In addition, a longer duration of hyperglycemia could impose prolonged damage to the glial and neuronal functions, leading to higher glaucoma risk [4].

Studies of the association of blood pressure with risk of glaucoma have not been entirely consistent. In a pooled analysis, every 10 mmHg increase in SBP was associated with a 1% increased risk of POAG, while every 5 mmHg increase in DBP was associated a 2% increased risk of POAG [9]. However, a multi-cohort analysis reported limited genetic correlation between DBP and POAG [31]. Another updated meta-analysis further found that people who have an unstable DBP, either high or low, are both able to increase the risk of OAG events [10]. Systemic hypertension may contribute to increased intraocular pressure (IOP) via overproduction or impaired outflow of aqueous humor [32,33]. Although hypertension has been reported as a risk factor in observational studies, our study may be the first to evaluate the causal association of blood pressure with glaucoma by using MR analysis. We found that SBP was suggestively associated with the risk of glaucoma, while DBP was not. Our finding was corroborated with a recent prospective study that showed an increased systolic blood pressure was associated with a greater risk of glaucoma related traits [34]. Further investigation is needed to validate our findings.

With respect to blood lipids, consistent associations with risk of glaucoma have been found in three meta-analyses, reporting that higher cholesterol, triglyceride, and LDL levels, as well as lower HDL levels, were associated with increased risk of glaucoma [6,7,8]. Although these findings supported the hypothesis that lipid levels posed an additional risk for glaucoma development, heterogeneity was substantial, and causality could not be presumed from identified observational studies. Few studies have reported the association between obesity and glaucoma, and the results remained controversial, with individual studies suggesting positive [11], inverse [35], and null [12] associations. In a recent MR study with 1824 POAG cases from database and 31 BMI SNPs, a positive association between BMI and POAG was found [36]. In this MR study that included larger glaucoma cases (20,906) and an updated SNP set, we found null patterns of associations for blood lipids and BMI with risk of glaucoma and a consistency of these associations in two populations, indicating that our results may have afforded these analyses greater instrument strength and greater statistical power to detect effects. However, we still emphasize that independent GWAS and large prospective studies are warranted in the further study.

The major strengths of the present study are the MR design, the large number of glaucoma cases, and the systematic assessment of multiple metabolic traits. Replicated results in two independent populations supported the robustness and reliability of our results for the associations of T2D and related traits with glaucoma. The meta-analysis of two data sources also increased the power to detect weak associations. Results from several sensitivity analyses further guaranteed the robustness of our findings. Most of the participants in our analyses were of European ancestry, thereby diminishing stratification bias.

Limitations in our study warrant consideration. First, although consistent results were observed using multiple sensitivity analyses, the pleiotropy could not be completely ruled out, which was a potential limitation in any MR study. Second, the definition of glaucoma in the UK Biobank relied on self-report, thus misdiagnosis bias may have occurred. Third, in the present study, we studied glaucoma as an entirety. Nevertheless, glaucoma includes diverse subtypes with different pathologies. Separate analyses for each of the disease subtype in the future would help clarify the difference in their associations with metabolic traits and lifestyle factors. Fourth, the use of publicly available summary-level data precluded us from assessing nonlinear associations. Fifth, the population confinement might limit the generalizability of our findings to other populations, so that further validations are needed in other cohorts and ethnicities. Moreover, it is also necessary to carry out other prospective studies to validate the aspects related to the causality between glaucoma and dyslipidemia or obesity, as well as the association with lifestyle.

In conclusion, this study strengthens the causal inference that T2D and related traits (including FG and HbAc1) are possible risk factors for glaucoma. In addition, higher SBP may also increase the risk of glaucoma. No evidence was found for BMI, DBP, or blood lipids with glaucoma development. These findings have clinical and public health implications, as metabolic traits can be intervened earlier and easily.

## Figures and Tables

**Table 1:** Overview of the data sources used in the Mendelian randomization study.

Exposure or Outcome	Unit	Participants Included in Analysis	Adjustments	IVs ^a^	PMID
BMI	SD of BMI	806,834 European-descent individuals	Age, age square, sex, and 1–5 PCs	613	30239722
Systolic blood pressure	10 mmHg	757,601 European-descent individuals	Sex, age, age square, BMI, genotyping chips	227	30224653
Diastolic blood pressure	10 mmHg	757,602 European-descent individuals	Sex, age, age square, BMI, genotyping chips	292	30224653
LDL cholesterol	SD of LDL cholesterol	188,578 individuals of multiancestries (90% European)	Age, age square, sex	80	24097068
HDL cholesterol	SD of HDL cholesterol	188,578 individuals of multiancestries (90% European)	Age, age square, sex	87	24097068
Triglyceride	SD of Triglyceride	188,578 individuals of multiancestries (90% European)	Age, age square, sex	55	24097068
Total cholesterol	SD of Total cholesterol	188,578 individuals of multiancestries (90% European)	Age, age square, sex	86	24097068
Fasting glucose	mmol/L	200,622 European-descent individuals	BMI, study-specific covariates, and PCs	69	34059833
Fasting insulin	pmol/L	151,013 European-descent individuals	BMI, study-specific covariates, and PCs	36	34059833
Hemoglobin A1c	1%	146,806 European-descent individuals	study-specific covariates and PCs	76	34059833
Type 2 diabetes	1-log unit odds of type 2 diabetes	62,892 type 2 diabetes cases and 596,424 controls of European ancestry	Age, sex, and 20 PCs	135	30054458
Glaucoma (UKBB + GERA)	—	12,315 glaucoma cases and 227,987 noncases of multiancestries (89% European)	Age, sex, and ancestry PCs	—	29891935
Glaucoma (FinnGen)	—	8591 glaucoma cases and 210,201 noncases of European descent	Age, sex, 10 PCs, and genotyping batch	—	—

Note: BMI, body mass index; IVs, instrument variables; HDL, high-density lipoprotein; LDL, low-density lipoprotein; PCs, principal components; SD, standard deviation. ^a^ Instrument variables used in the present Mendelian randomization analysis.

## Data Availability

The data presented in this study are available in article and supplementary material.

## Supplementary Materials

The following supporting information can be downloaded at: https://www.mdpi.com/article/10.3390/metabo13010109/s1, Table S1: Sensitivity analyses of genetically predicted risk factors with glaucoma in the meta-analysis of the UK Biobank and GERA studies; Table S2: Sensitivity analyses of genetically predicted risk factors with glaucoma in the FinnGen consortium; Table S3: Multivariable Mendelian randomization associations of genetically predicted type 2 diabetes with glaucoma adjusted for confounding traits.

## Abbreviations

BMI: body mass index; CI, confidence interval; DBP, diastolic blood pressure; FI, fasting insulin; FG, fasting glucose; GERA, Genetic Epidemiology Research in Adult Health and Aging; GWASs, genome-wide association studies; HbA1c, hemoglobin A1c; HDL, high-density lipoprotein; IOP, intraocular pressure; IVs, instrumental variables; LDL, low-density lipoprotein; MR, Mendelian randomization; MR-PRESSO, Mendelian randomization pleiotropy RESidual sum and outlier; OR, odds ratio; OAG, open-angle glaucoma; POAG, primary open-angle glaucoma; SBP, systolic blood pressure; SNPs, single nucleotide polymorphisms; T2D, type 2 diabetes; TC, total cholesterol; TG, triglyceride; UKBB, UK Biobank.

## References

[1] Tham Y.C., Li X., Wong T.Y., Quigley H.A., Aung T., Cheng C.Y. (2014). Global prevalence of glaucoma and projections of glaucoma burden through 2040: A systematic review and meta-analysis. Ophthalmology.

[2] Quigley H.A. (2011). Glaucoma. Lancet.

[3] Gumus M., Babacan S.N., Demir Y., Sert Y., Koca I., Gulcin I. (2022). Discovery of sulfadrug-pyrrole conjugates as carbonic anhydrase and acetylcholinesterase inhibitors. Arch. Pharm..

[4] Zhao D., Cho J., Kim M.H., Friedman D.S., Guallar E. (2015). Diabetes, fasting glucose, and the risk of glaucoma: A meta-analysis. Ophthalmology.

[5] Zhao Y.X., Chen X.W. (2017). Diabetes and risk of glaucoma: Systematic review and a Meta-analysis of prospective cohort studies. Int. J. Ophthalmol..

[6] Pertl L., Mossbock G., Wedrich A., Weger M., Konigsbrugge O., Silbernagel G., Posch F. (2017). Triglycerides and Open Angle Glaucoma—A Meta-analysis with meta-regression. Sci. Rep..

[7] Posch-Pertl L., Michelitsch M., Wagner G., Wildner B., Silbernagel G., Pregartner G., Wedrich A. (2022). Cholesterol and glaucoma: A systematic review and meta-analysis. Acta Ophthalmol..

[8] Wang S., Bao X. (2019). Hyperlipidemia, Blood Lipid Level, and the Risk of Glaucoma: A Meta-Analysis. Investig. Ophthalmol. Vis. Sci..

[9] Zhao D., Cho J., Kim M.H., Guallar E. (2014). The association of blood pressure and primary open-angle glaucoma: A meta-analysis. Am. J. Ophthalmol..

[10] Nislawati R., Taufik Fadillah Zainal A., Ismail A., Waspodo N., Kasim F., Gunawan A. (2021). Role of hypertension as a risk factor for open-angle glaucoma: A systematic review and meta-analysis. BMJ Open Ophthalmol..

[11] Ko F., Boland M.V., Gupta P., Gadkaree S.K., Vitale S., Guallar E., Zhao D., Friedman D.S. (2016). Diabetes, Triglyceride Levels, and Other Risk Factors for Glaucoma in the National Health and Nutrition Examination Survey 2005–2008. Investig. Ophthalmol. Vis. Sci..

[12] Cheung N., Wong T.Y. (2007). Obesity and eye diseases. Surv. Ophthalmol..

[13] Laville V., Kang J.H., Cousins C.C., Iglesias A.I., Nagy R., Cooke Bailey J.N., Igo R.P., Song Y.E., Chasman D.I., Christen W.G. (2019). Genetic Correlations Between Diabetes and Glaucoma: An Analysis of Continuous and Dichotomous Phenotypes. Am. J. Ophthalmol..

[14] Pulit S.L., Stoneman C., Morris A.P., Wood A.R., Glastonbury C.A., Tyrrell J., Yengo L., Ferreira T., Marouli E., Ji Y. (2019). Meta-analysis of genome-wide association studies for body fat distribution in 694 649 individuals of European ancestry. Hum. Mol. Genet..

[15] Evangelou E., Warren H.R., Mosen-Ansorena D., Mifsud B., Pazoki R., Gao H., Ntritsos G., Dimou N., Cabrera C.P., Karaman I. (2018). Genetic analysis of over 1 million people identifies 535 new loci associated with blood pressure traits. Nat. Genet..

[16] Willer C.J., Schmidt E.M., Sengupta S., Peloso G.M., Gustafsson S., Kanoni S., Ganna A., Chen J., Buchkovich M.L., Mora S. (2013). Discovery and refinement of loci associated with lipid levels. Nat. Genet..

[17] Chen J., Spracklen C.N., Marenne G., Varshney A., Corbin L.J., Luan J., Willems S.M., Wu Y., Zhang X., Horikoshi M. (2021). The trans-ancestral genomic architecture of glycemic traits. Nat. Genet..

[18] Xue A., Wu Y., Zhu Z., Zhang F., Kemper K.E., Zheng Z., Yengo L., Lloyd-Jones L.R., Sidorenko J., Wu Y. (2018). Genome-wide association analyses identify 143 risk variants and putative regulatory mechanisms for type 2 diabetes. Nat. Commun..

[19] Clarke L., Zheng-Bradley X., Smith R., Kulesha E., Xiao C., Toneva I., Vaughan B., Preuss D., Leinonen R., Shumway M. (2012). The 1000 Genomes Project: Data management and community access. Nat. Methods.

[20] Yang F., Chen S., Qu Z., Wang K., Xie X., Cui H. (2021). Genetic Liability to Sedentary Behavior in Relation to Stroke, Its Subtypes and Neurodegenerative Diseases: A Mendelian Randomization Study. Front. Aging Neurosci..

[21] Choquet H., Paylakhi S., Kneeland S.C., Thai K.K., Hoffmann T.J., Yin J., Kvale M.N., Banda Y., Tolman N.G., Williams P.A. (2018). A multiethnic genome-wide association study of primary open-angle glaucoma identifies novel risk loci. Nat. Commun..

[22] Track Football Consortium (2021). FinnGen Documentation of R5 Release. https://finngen.gitbook.io/documentation/.

[23] Bowden J., Davey Smith G., Haycock P.C., Burgess S. (2016). Consistent Estimation in Mendelian Randomization with Some Invalid Instruments Using a Weighted Median Estimator. Genet. Epidemiol..

[24] Burgess S., Thompson S.G. (2017). Interpreting findings from Mendelian randomization using the MR-Egger method. Eur. J. Epidemiol..

[25] Hemani G., Zheng J., Elsworth B., Wade K.H., Haberland V., Baird D., Laurin C., Burgess S., Bowden J., Langdon R. (2018). The MR-Base platform supports systematic causal inference across the human phenome. Elife.

[26] Verbanck M., Chen C.Y., Neale B., Do R. (2018). Detection of widespread horizontal pleiotropy in causal relationships inferred from Mendelian randomization between complex traits and diseases. Nat. Genet..

[27] Burgess S., Thompson S.G. (2015). Multivariable Mendelian randomization: The use of pleiotropic genetic variants to estimate causal effects. Am. J. Epidemiol..

[28] Shen L., Walter S., Melles R.B., Glymour M.M., Jorgenson E. (2016). Diabetes Pathology and Risk of Primary Open-Angle Glaucoma: Evaluating Causal Mechanisms by Using Genetic Information. Am. J. Epidemiol..

[29] Kanamori A., Nakamura M., Mukuno H., Maeda H., Negi A. (2004). Diabetes has an additive effect on neural apoptosis in rat retina with chronically elevated intraocular pressure. Curr. Eye Res..

[30] Nakamura M., Kanamori A., Negi A. (2005). Diabetes mellitus as a risk factor for glaucomatous optic neuropathy. Ophthalmologica.

[31] Aschard H., Kang J.H., Iglesias A.I., Hysi P., Cooke Bailey J.N., Khawaja A.P., Allingham R.R., Ashley-Koch A., Lee R.K., Moroi S.E. (2017). Genetic correlations between intraocular pressure, blood pressure and primary open-angle glaucoma: A multi-cohort analysis. Eur. J. Hum. Genet..

[32] He Z., Vingrys A.J., Armitage J.A., Bui B.V. (2011). The role of blood pressure in glaucoma. Clin. Exp. Optom..

[33] Wu X., Konieczka K., Liu X., Chen M., Yao K., Wang K., Flammer J. (2022). Role of ocular blood flow in normal tension glaucoma. Adv. Ophthalmol. Pract. Res..

[34] Marshall H., Mullany S., Qassim A., Siggs O., Hassall M., Ridge B., Nguyen T., Awadalla M., Andrew N.H., Healey P.R. (2021). Cardiovascular Disease Predicts Structural and Functional Progression in Early Glaucoma. Ophthalmology.

[35] Ramdas W.D., Wolfs R.C., Hofman A., de Jong P.T., Vingerling J.R., Jansonius N.M. (2011). Lifestyle and risk of developing open-angle glaucoma: The Rotterdam study. Arch. Ophthalmol..

[36] Lin Y., Zhu X., Luo W., Jiang B., Lin Q., Tang M., Li X., Xie L. (2022). The Causal Association Between Obesity and Primary Open-Angle Glaucoma: A Two-Sample Mendelian Randomization Study. Front. Genet..

