# Supplementary material for: Appraising the Effects of Metabolic Traits on the Risk of Glaucoma: A Mendelian Randomization Study
Source: Metabolites. 2023 Jan 9;13(1):109. doi: 10.3390/metabo13010109 (PMC9867384; doi:10.3390/metabo13010109)
Supplement: Supplementary file 1 [file metabolites-13-00109-s001.zip › metabolites-2096394-supplementary.pdf]

Table S1. Sensitivity analyses of genetically predicted risk factors with glaucoma in the meta-analysis of the UK Biobank and GERA studies.

			Weighted Median		MR-Egger			Maximum Likelihood		MR-PRESSO			
Exposures	Used SNPs	Cochrane's Q	OR (95% CI)	*P*	OR (95% CI)	*P*	$P_{intercept}$[a]	OR (95% CI)	*P*	Outliers	OR (95% CI)	*P*	$P_{distortion}$[b]
BMI	606	876	1.09 (0.95, 1.24)	0.237	1.33 (1.04, 1.71)	0.026	0.027	1.02 (0.95, 1.10)	0.584	6	1.00 (0.92, 1.10)	0.930	0.073
SBP	225	404	0.99 (0.87, 1.11)	0.826	0.83 (0.64, 1.06)	0.131	0.054	1.04 (0.97, 1.11)	0.323	5	1.03 (0.95, 1.13)	0.451	0.953
DBP	287	464	1.06 (0.89, 1.26)	0.511	0.78 (0.57, 1.08)	0.132	0.066	1.03 (0.93, 1.14)	0.563	4	1.01 (0.89, 1.14)	0.897	0.130
LDL-C	77	114	1.02 (0.92, 1.14)	0.686	0.98 (0.87, 1.11)	0.798	0.823	0.99 (0.93, 1.06)	0.871	2	0.99 (0.93, 1.07)	0.874	0.993
HDL-C	87	129	0.99 (0.87, 1.12)	0.877	0.93 (0.79, 1.10)	0.391	0.559	0.97 (0.90, 1.04)	0.404	0	NA	NA	NA
TG	55	95	0.94 (0.83, 1.06)	0.307	0.95 (0.80, 1.13)	0.587	0.775	0.93 (0.86, 1.01)	0.102	1	0.95 (0.87, 1.03)	0.241	0.608
TC	83	130	0.99 (0.89, 1.10)	0.804	0.95 (0.83, 1.08)	0.410	0.288	1.00 (0.94, 1.07)	0.960	2	1.01 (0.94, 1.09)	0.778	0.878
FG	68	128	1.37 (1.05, 1.78)	0.020	1.49 (0.99, 2.25)	0.063	0.364	1.27 (1.08, 1.50)	0.004	1	1.30 (1.04, 1.61)	0.022	0.837
FI	34	67	0.86 (0.52, 1.40)	0.536	0.82 (0.18, 3.74)	0.798	0.769	1.02 (0.73, 1.42)	0.912	2	1.00 (0.68, 1.48)	0.998	0.004
HbA1c	72	108	1.50 (1.03, 2.20)	0.036	1.22 (0.73, 2.05)	0.446	0.770	1.31 (1.04, 1.65)	0.021	2	1.30 (1.00, 1.68)	0.051	0.976
T2D	134	433	1.06 (1.00, 1.13)	0.061	1.07 (0.92, 1.25)	0.399	0.933	1.08 (1.04, 1.12)	1.1×10^{-4}	6	1.05 (1.00, 1.09)	0.050	0.150

Note: BMI, body mass index; CI, confidence intervals; DBP, diastolic blood pressure; FG, fasting glucose; FI, fasting insulin; HbA1c, hemoglobin A1c; HDL-C, high-density lipoprotein cholesterol; LDL-C, low-density lipoprotein cholesterol; NA, not available; OR, odds ratio; SBP, systolic blood pressure; T2D, type 2 diabetes; TC, total cholesterol; TG, triglyceride.

[a] $P < 0.05$ indicates a statistically significant pleiotropic effect MR-Egger intercept test.

[b] $P < 0.05$ indicates a statistically significant difference between estimates before and after outlier removal in MR-PRESSO test.

Table S2. Sensitivity analyses of genetically predicted risk factors with glaucoma in the FinnGen consortium.

			Weighted Median		MR-Egger			Maximum Likelihood		MR-PRESSO			
Exposures	Used SNPs	Cochrane's Q	OR (95% CI)	*P*	OR (95% CI)	*P*	$P_{intercept}$[a]	OR (95% CI)	*P*	Outliers	OR (95% CI)	*P*	$P_{distortion}$[b]
BMI	600	688	1.01 (0.86, 1.19)	0.892	1.19 (0.90, 1.58)	0.219	0.063	0.93 (0.84, 1.03)	0.149	4	0.93 (0.84, 1.02)	0.134	0.924
SBP	216	326	1.13 (0.97, 1.32)	0.103	1.02 (0.77, 1.37)	0.869	0.436	1.14 (1.04, 1.25)	0.004	1	1.13 (1.02, 1.26)	0.025	0.859
DBP	273	387	0.99 (0.79, 1.23)	0.913	1.12 (0.78, 1.62)	0.542	0.740	1.06 (0.93, 1.20)	0.370	1	1.07 (0.93, 1.25)	0.345	0.888
LDL-C	78	97	1.03 (0.91, 1.17)	0.648	1.10 (0.97, 1.23)	0.131	0.132	1.03 (0.95, 1.10)	0.488	0	NA	NA	NA
HDL-C	86	105	0.98 (0.84, 1.14)	0.807	1.06 (0.88, 1.27)	0.535	0.089	0.93 (0.85, 1.01)	0.094	0	NA	NA	NA
TG	53	70	0.93 (0.79, 1.08)	0.335	0.93 (0.77, 1.13)	0.498	0.802	0.95 (0.86, 1.06)	0.366	1	0.97 (0.87, 1.07)	0.539	0.501
TC	82	88	1.03 (0.92, 1.16)	0.598	1.03 (0.90, 1.18)	0.646	0.698	1.01 (0.93, 1.09)	0.792	0	NA	NA	NA
FG	65	96	1.23 (0.90, 1.69)	0.190	1.12 (0.71, 1.75)	0.637	0.656	1.22 (0.99, 1.50)	0.064	1	1.28 (1.02, 1.62)	0.041	0.675
FI	35	90	0.81 (0.43, 1.50)	0.494	0.90 (0.10, 7.96)	0.928	0.818	1.16 (0.77, 1.76)	0.475	3	0.82 (0.48, 1.39)	0.465	0.249
HbA1c	72	132	1.31 (0.81, 2.11)	0.276	1.50 (0.69, 3.28)	0.313	0.542	1.22 (0.90, 1.65)	0.192	3	1.27 (0.89, 1.80)	0.198	0.849
T2D	133	213	1.19 (1.09, 1.29)	5.6×10^{-05}	1.15 (1.00, 1.33)	0.058	0.869	1.14 (1.09, 1.20)	5.0×10^{-8}	3	1.13 (1.07, 1.19)	1.1×10^{-5}	0.678

Note: BMI, body mass index; CI, confidence intervals; DBP, diastolic blood pressure; FG, fasting glucose; FI, fasting insulin; HbA1c, hemoglobin A1c; HDL-C, high-density lipoprotein cholesterol; LDL-C, low-density lipoprotein cholesterol; NA, not available; OR, odds ratio; SBP, systolic blood pressure; T2D, type 2 diabetes; TC, total cholesterol; TG, triglyceride.

[a] $P < 0.05$ indicates a statistically significant pleiotropic effect MR-Egger intercept test.

[b] $P < 0.05$ indicates a statistically significant difference between estimates before and after outlier removal in MR- PRESSO test.

Table S3. Multivariable Mendelian randomization associations of genetically predicted type 2 diabetes with glaucoma adjusted for confounding traits.

	UK Biobank + GERA		FinnGen consortium		Combined	
Model	OR (95% CI)	*P*	OR (95% CI)	*P*	OR (95% CI)	*P*
Unadjusted model	1.08 (1.01, 1.15)	0.033	1.14 (1.07, 1.21)	2.0×10^{-5}	1.11 (1.06, 1.16)	4.4×10^{-6}
Adjusted for BMI	1.07 (1.00, 1.15)	0.040	1.14 (1.07, 1.21)	2.9×10^{-5}	1.11 (1.06, 1.16)	7.9×10^{-6}
Adjusted for SBP	1.09 (1.01, 1.17)	0.030	1.12 (1.05, 1.20)	1.0×10^{-3}	1.11 (1.05, 1.16)	8.9×10^{-5}
Adjusted for DBP	1.08 (1.01, 1.16)	0.022	1.14 (1.07, 1.22)	2.8×10^{-5}	1.12 (1.07, 1.17)	3.0×10^{-6}
Adjusted for LDL-C	1.07 (1.00, 1.15)	0.049	1.14 (1.07, 1.22)	4.6×10^{-5}	1.11 (1.06, 1.16)	1.5×10^{-5}
Adjusted for HDL-C	1.09 (1.01, 1.17)	0.024	1.15 (1.07, 1.23)	5.8×10^{-5}	1.12 (1.07, 1.18)	6.7×10^{-6}
Adjusted for TG	1.08 (1.01, 1.16)	0.031	1.14 (1.07, 1.22)	7.2×10^{-5}	1.11 (1.06, 1.17)	1.2×10^{-5}
Adjusted for TC	1.07 (1.00, 1.15)	0.049	1.14 (1.07, 1.22)	4.0×10^{-5}	1.11 (1.06, 1.16)	1.6×10^{-5}
Adjusted for FG	1.06 (0.98, 1.16)	0.159	1.15 (1.06, 1.24)	5.9×10^{-4}	1.11 (1.05, 1.17)	4.6×10^{-4}
Adjusted for FI	1.08 (1.01, 1.15)	0.028	1.14 (1.07, 1.21)	3.2×10^{-5}	1.11 (1.06, 1.16)	5.6×10^{-6}
Adjusted for HbA1c	1.06 (0.99, 1.13)	0.127	1.12 (1.06, 1.20)	2.5×10^{-4}	1.09 (1.04, 1.14)	2.0×10^{-4}
Adjusted for smoking	1.07 (1.00, 1.15)	0.039	1.14 (1.07, 1.21)	2.9×10^{-5}	1.11 (1.06, 1.16)	7.7×10^{-6}
Adjusted for alcohol	1.07 (1.00, 1.15)	0.039	1.13 (1.06, 1.20)	1.2×10^{-4}	1.10 (1.06, 1.16)	2.2×10^{-5}

Note: BMI, body mass index; CI, confidence intervals; DBP, diastolic blood pressure; FG, fasting glucose; FI, fasting insulin; HbA1c, hemoglobin A1c; HDL-C, high-density lipoprotein cholesterol; LDL-C, low-density lipoprotein cholesterol; OR, odds ratio; SBP, systolic blood pressure; TC, total cholesterol; TG, triglyceride.
